# An Account of Remarkable Effects from the Application of the Actual Cautery to the Nape of the Neck and the Top of the Head, in Several Diseases, Both Idiopathic and Symptomatic, of the Eyes, Ears and Brain

**Published:** 1808-05

**Authors:** Louis Valentin

**Affiliations:** formerly Professor of Anatomy at Cape-Francois, &c. &c. now of Nancy, in Lorraine


### *An* ACCOUNT *of* REMARKABLE EFFECTS *from the* APPLICATION *of the* ACTUAL CAUTERY *to the* NAPE *of the* NECK *and the* TOP *of the* HEAD, *in several Diseases, both Idiopathic and Symptomatic, of the Eyes, Ears and Brain. By* LOUIS VALENTIN, M. D. *formerly Professor of Anatomy at Cape-Francois, &c. &c. now of Nancy, in Lorraine. Communicated to Dr.* MITCHILL, *of New York.*

### SECTION I.

IT is well known how much the actual cautery was used by the ancients; and, at this day, great is the extent of the practice among the Egyptians, the Japanese and Chinese, either by the *moxa* or the hot iron, for pains of almost almost all parts of the body. It was so familiar with the Grecians and Scythians, that they looked upon diseases as incurable when fire had not conquered them. Thereupon Hippocrates said: *Quod remedium non sanat ferrum sanat, quod ferum non sanat ignis sanat, quod ignis non sanat insanabile dici oportet.*" This excellent method has been renewed and celebrated in our age, by Pouteau of Lyons.* I do not mean to relate all the cases where I have successfully employed this help in the healing art, but shall confine my remarks merely to what concerns the principal diseases of the head and face, as it has been extensively used in them.

### Section II.

In many kinds of obstinate ophthalmias, in the blindness that follows them, and in serious old sores of the eyes, whatever the cause might be, in which all means, both internal and external, blisters and setons on the hinder part of the neck, had been employed, I have always observed the efficacy of fire upon the top of the head to deserve a preference. It must be applied about the middle of the hinder edge of the coronal bone (the place being shaved) and the extent of the burning at least as large as a dollar The application produces, indeed, an acute pain, but the patients are greatly relieved by the useful effects they derive from it. The part must be immediately anointed with oil, and covered with a soft and suppurative plaster. The next day two or three crucial incisions must be made into the burned spot, in order to accelerate the suppuration, which becoming more copious, and being nearer the seat of the disorders than when these emunctories are put on the nape or occipital region, they produce a speedier relief; besides the primitive effects of the exustion, in changing the nervous irradiations, and attracting, as about a centre, the humours of the head. I prefer it to the other external irritants, as it maintains for a longer time a copious discharge of matter.

At Cape-François, where sore eyes aro common and very obstinate, and their cure very difficult, I have cured many people by this salutary method, though they had already taken advice from all parts, and tried, unsuccessfully, various kinds of remedies, for a long time; and this

* See the posthumous works of that ingenious and skilful surgeon, by Du Colombier, of Paris. Prosper Alpin. Med. des Egyptiens. Du Jardin, Histoire de la Chirurgie, tom. i. à Paris.

this even when the blindness was acknowledged and certified by professional men.*

I oftentimes scarified and took off portions of the tunica conjunctiva, when the vessels were considerably choaked up, particularly iu the *opthalmia chemosis, opthalmia ulcerosa,* and many others. In all cases one or two pukes must be administered previous to the cauterization, and they should be used rather than purges.† The softening and lenient topicks and collyria ought to be preferred to those which are acrid and irritating, in the increase as well as in the decrease of the disease.

### Section III.

'In sore ears, as the *otalgia inflammatoria,* accompanied with delirium, convulsions or drowsiness, when liniments, topicks, cupping-glasses, leeches, or blisters, have been unsuccessfully employed, the hot iron behind the ear, upon the *processus mastoideus,* succeeds perfectly well. I have also cauterized advantageously, the inward portion of the ear with a smaller red iron, slipping through a sheath, with a design of appeasing tooth-ache, and pains of the jaws and the contiguous parts, produced by a defluxion to that region, as some of our predecessors have done.

### Section IV.

I have seen it likewise produce good effects in acute and obstinate pains of the head—that is to say, in *cephalalgia* and *cephalæa,* as well as in madness, after an unsuccessful use of other means. Then it is better to cauterize the vertex strongly, and, if it be possible, on the painful place, particularly when the fits return periodically, and have not been sufficiently overcome by the red-powder-bark.

Some have also employed a large blister-plaster, covering all the head; and in malignant fevers and commotions of the

* A fact the most known and desperate concerns a young lieutenant of volunteers, whose cure the general assembly of the colony had trusted to me, about the beginning of the year 1792, after the exhibition of a certificate delivered to him by the two first health-officers of the charity-hospital, where he had before been treated, attesting the loss of his sight, and his being passed as incurable. The proof of the radical cure is mentioned in the verbal procés of that assembly, June 24, 1792. The patient recovered his sight in less than five weeks.

† In general, I have observed, in all cases of sore eyes, that pukes succeed better than purges. This preference given to pukes, in diseases seated above the diaphragm, agrees with the aphorism of Hippocrates: *Quæ, supra septum transversum vomitus, et infra purgationes requirunt.*

the brain its effects appear very salutary.* Others have cauterized the head to the very skull, or even to the bone itself, after having cut away the hairy scalp, in madness and in epilepsy.† But these experiments were not always prosperous.

SECTION V.

It is in phrenitis, in the frightful symptoms of putrid and malignant fevers, and others of an eruptive kind, or coma, delirium, convulsive motions, *prostratio virium, &c.* that the applications of the actual cautery on the occiput is very useful. I could give an account of many remarks made in France, in the West Indies, and particularly in Virginia, with regard to the hot iron in the treatment of the above diseases. I shall confine myself, however, to a few facts.

Mademoiselle Pellier, a superannuated maid, at Nancy, in Lorraine, living near the Botanical Garden, was attacked by an excessive phrenitis, in the year 1787, after the operation of a violent purge she had taken for the head and belly-ache. She was furious, and they were obliged to tie her in bed. Her pulse was low and frequent, and her eyes animated. She constantly refused swallowing. Repeated employment of the semicupium, and blisters put on the legs, dry frictions, cooling topics upon the head, were inefficacious, and nothing could appease or relieve her. Having been consulted on her dangerous case, I cauterized the nape of the neck without delay. Her great agitation, flow of words, and cries, ceased immediately. She remained with her eyes open, and speechless, during two hours; after which shd slept till the next morning. Her fever was moderate, her pulse stronger and more free, and that disease which had a putrid character was radically cured in a fortnight.

SCECTION VI.

At St. Domingo, where malignant fevers of a bad character, agues, and intermitting fevers, are exceedingly common amongst the white people, the cauterization of the nape of the neck, precisely between the occipital bone and the first cervical vertebra, near the superior insertions of the trapezius muscle, has very often succeeded with me when

* The blister-plaster, in the form of a cap, is known to be useful in commotions of the brain; and M. Desault, chief surgeon of the Hotel Dieu, at Paris, has used it frequently, and with success.

† Vide Dehaen, ratio medendi.

when the sick had fallen into a coma, with convulsive motions, and a closing of the jaws. Sometimes the power of swallowing was lost, besides shortness of breath, &c. If the patient is depressed, low, and sinking under the disease, notwithstanding blisters and the use of quinquina, so advantageous in this state; if the nervous system was a little affected, the brain confused, and the mind roving, and those means were inadequate to put a stop to these disorders, I apply the burning iron without delay. I made use of it early, according to the emergency, but never before having obtained the necessary evacuations; it would otherwise be useless. It may be employed at any other times, towards the sixteenth, seventeenth, or twenty-first day; seldom later in those climates.

Two young men in Cape Francois underwent this operation at an advanced period of their sickness, after which the crisis* and cure appeared to take place. One, named Vetcon (chevalier) fell suddenly senseless on the seventeenth day: the teeth closed, the belly swelled, and he was rapidly declining under his symptoms, though he had all along received regular treatment and attendance. He at length threw up all his medicines.—The other, named Arnold, a watchmaker, from Nancy, was struck with the same desperate symptoms on the sixteenth day; having had, till that time, a simple remittent fever, regularly treated. Convulsions and dreadful howlings, affrighting the neighbourhood and the people passing by, not being abated by cold water poured on the head, blisters on the legs, assisted by sinapisms, &c. I fully cauterized the occipit. He soon began to swallow some spoonfuls of antispamodic mixture and powder-bark; and the disease was happily terminated, by degrees, in the former about the thirty-first day, and in the latter the twenty-second.

### Section VII.

The same operation was very advantageous to us in the fatal epidemic disease which afflicted the seamen of the French squadron which was at anchor in Chesapeake-Bay in the months of February, March and April, of the year 1794. These men, miserably distempered, were placed in

* Those diseases are commonly shorter in the Antilles than elsewhere: in general, the crises are never observed, because of the early and so amazingly profitable use of the quinquina.

our three hospitals, established on both sides of Elizabeth River, near Norfolk.

The character of the disorder was a putrid and malignant fever. Some were troubled with worms and peripneumony. The greatest part were brought to me in a state of debility and despondency—being often in a delirium—having a blackish looseness, little fever, and scarcely sixty-eight or seventy-two pulsations in a minute. There were some in whom the vital strength languished so much, that I found only thirty-six, forty-four, forty-eight, and now and then from fifty to seventy, pulsations in a minute.

Others, very much animated, and stronger, having a full and frequent pulse, became soon frantic, and getting out of their beds, wandered up and down, and obliged us tie them. For some of these I prescribed bleeding, with anodyne and antispamodic remedies. Sometimes they refused them. But nothing abated those symptoms better than cauterizing. They recovered sooner and more easily than the former, to whom this operation was made in the state of delirium and of tremours in the extremities. Blister plasters and quinquina kept almost an equal pace together: sometimes Huxham's tincture, with camphor and vitriolic acid.

Many of those patients underwent the hot iron. One day, in my morning visit, I cauterized eleven of them in a single hospital, and we were satisfied that many owe their salvation to it. Fire has two effects: 1. In awakening and reanimating the benumbed sensitive principle in the former, or the weakest patients; in changing the nervous irradiations more suddenly, and having a tendency to destroy the other irradiation so hurtful to the system, &c. in the latter. 2. By determining, in both, a more copious flow of humours from the tela cellulosa (since the skin is disorganized), and duriug a longer time than can be effected by blisters.

We saw some parotids, but almost all were mortal, notwithstanding the stimulating topicks. The cautery applied to the tumours was more beneficial. At length, attendance failing, many died, because the health-officers, as well as myself, inhaled the poisonous vapour, and sickened in consequence thereof.

